# Differences of endogenous polyamines and putative genes associated with paraquat resistance in goosegrass (*Eleusine indica L*.)

**DOI:** 10.1371/journal.pone.0216513

**Published:** 2019-12-26

**Authors:** Qiyu Luo, Jiping Wei, Zhaoxia Dong, Xuefeng Shen, Yong Chen

**Affiliations:** Department of Crop Cultivation and Farming System, South China Agricultural University, Guangzhou, Guangdong, China; University of Melbourne, AUSTRALIA

## Abstract

**Background:**

Paraquat is one of the most effective herbicides used to control weeds in agricultural management, while the pernicious weed goosegrass (*Eleusine indica*) has evolved resistance to herbicides, including paraquat. Polyamines provide high-level paraquat resistance in many plants. In the present study, we selected three polyamines, namely, putrescine, spermidine, and spermine, as putative genes to investigate their correlation with paraquat resistance by using paraquat-resistant (R) and paraquat-susceptible (S) goosegrass populations.

**Results:**

There was no significant difference in the putrescine nor spermine content between the R and S biotypes. However, 30 and 90 min after paraquat treatment, the spermidine concentration was 346.14-fold and 421.04-fold (P < 0.001) higher in the R biotype than in the S biotype, but the spermidine concentration was drastically reduced to a marginal level after 90 min. Since the transcript level of *PqE* was low while the spermidine concentration showed a transient increase, the *PqE* gene was likely involved in the synthesis of the paraquat resistance mechanism, regulation of polyamine content, and synthesis of spermidine and spermine. *PqTS1*, *PqTS2*, and *PqTS3* encode transporter proteins involved in the regulation of paraquat concentration but showed different transcription patterns with synchronous changes in polyamine content.

**Conclusion:**

Endogenous polyamines (especially spermidine) play a vital role in paraquat resistance in goosegrass. *PqE*, *PqTS1*, *PqTS2*, and *PqTS3* were speculated on the relationship between polyamine metabolism and paraquat resistance. To validate the roles of *PqE*, *PqTS1*, *PqTS2*, and *PqTS3* in polyamine transport systems, further research is needed.

## Introduction

Herbicides are the most widely used tool for weed management in large production areas where hand-weeding is neither efficient nor economical [1]. Paraquat is a haloid salt [2] that is widely used as a non-selective herbicide to control weeds throughout the world [3–5]. Paraquat resistance in plant cells is determined by the uptake, efflux, sequestration, detoxification, and catabolism of the reactive oxygen species (ROS) generated by paraquat [4]. Two hypotheses have been proposed for paraquat resistance in plants: One is an increase in the capability of plant cells to scavenge ROS, while the other is the ability of plant cells to sequester paraquat away from target sites in their chloroplasts [6].Although there are numerous data available on paraquat resistance in response to rapidly increasing multi-resistance in weeds threats against crop production worldwide, no cases of target site paraquat-resistance have been identified for its difficult metabolism [2,7]. Based on the review about mechanisms of paraquat-resistance weeds and model plants, mutative genes and enzymes can limit paraquat uptake in cytoplasm and chloroplasts to enhance tolerance but the molecular mechanism of these genes regulate high level paraquat-resistance in natural weeds have not been defined [8].

To date, nearly 30 species of R weed species have been reported worldwide [9]. Goosegrass (*Eleusine indica (L*.*)* Gaertn) is a pernicious weed species with a cosmopolitan distribution and is usually controlled by paraquat [10,11]. However, R biotypes of goosegrass have arisen due to the widespread use of the herbicide [12]. Our previous study identified 81 genes related to paraquat resistance in goosegrass. Fifty-three of these genes related to reactive oxygen species scavenging, but 10 of these genes were related to, polyamines, and the remaining 18 genes were related to polyamine transporters [13]. Thus, on this paper we make further research on the role of polyamine transporter genes in paraquat resistance in goosegrass can be used to understand the molecular mechanism of paraquat resistance.

Polyamines, including putrescine, spermidine and spermine, are small aliphatic polycations that are classified as growth regulators, although the specific mechanisms have not been well elucidated [14]. Exogenous application of polyamines provides high-level resistance against paraquat toxicity in a range of plant organs [5], such as the roots of intact maize seedlings [15], leaf discs of sunflower [16], and leaves of rice [17]. Genetic manipulation of anabolic and catabolic pathways has clearly elucidated that polyamine metabolism play an importance role in plant responses to exogenous stresses [18]. However, the transportation of polyamines in plant tissues remains much less known [19].

It is noteworthy that the structure of paraquat is similar to that of polyamines, and both can use the same transport system in their hosts [20]. Since polyamines can be transported into the vacuoles, a polyamine transporter likely mediates this transport process [21]. Quantification of the polyamine contents in paraquat-resistant (R) and paraquat-susceptible (S) *Arctotheca calendula* revealed a possible role of polyamines and/or polyamine transporters in paraquat resistance [22]. A study on the role of transporters in the paraquat resistance of horseweed showed that the 12-kDa *E*. *coli* multidrug transporter (EmrE) and an amino acid transporter (CAT4) were related to paraquat resistance, and the CAT4 transporter had affinity for substances with a charge distribution similar to that of polyamine molecules [2]. Despite extensive studies on polyamine metabolism, the functions of polyamine-related genes in plant resistance to paraquat remain unclear.

We focused on the genes *PqE*, *PqTS1*, *PqTS2*, and *PqTS3* on this paper, which were selected from 10 putative polyamines genes and 18 putative polyamine transporters genes. Because the genes *PqE*, *PqTS1*, *PqTS2*, and *PqTS3* are significantly upregulated in S and R biotypes 80 min after paraquat treatment, with filtered by DEGs and highly expressed goosegrass transcripts to get high resistant character values (fold change RQ/SQ) of +1.07, +1.01, +1.20, and +1.03, respectively. RQ comes from resistant goosegrass seedlings for mixed samples sprayed paraquat 40 min, 60 min and 80 min; SQ comes from susceptible goosegrass seedlings for mixed samples sprayed paraquat 40 min, 60 min and 80 min. Fold change is equal to log_2_ (RQ) [13]. Moreover, Transcriptome analysis revealed *PqE* as the putative gene related to polyamine synthesis, while *PqTS1*, *PqTS2*, and *PqTS3* were identified as the putative genes associated with paraquat resistance in goosegrass [13]. In this study, S and R biotypes of goosegrass were treated with paraquat for extending time to 180 min. Instead of using mixed samples sprayed paraquat 40, 60 and 80 min, we also specifically segregated samples sprayed paraquat from 0, 30, 60, 90, 120, and 180 min. We used real-time PCR (RT-PCR) to validate high-throughput sequencing data from each sample of S and R goosegrass, and the transcript levels of *PqE*, *PqTS1*, *PqTS2*, and *PqTS3* were determined after treatment. We also quantified endogenous polyamine contents in same samples of both biotypes using high-performance liquid chromatography (HPLC). Based on the results, we speculated on the relationship between the genes related to polyamine metabolism and paraquat resistance. These findings offer explanations of the molecular mechanisms of paraquat resistance.

## Materials and methods

### Plant materials and experimental treatments

The S and R biotypes including their seedling cultivation in this study keep the same as that in the previous transcriptome analysis paper [13]. The R biotype was collected from the Teaching and Research Farm (113°40´E, 22°80´N) in Panyu District, Guangzhou, China. Papaya (*Carica papaya* L.) and banana (*Musa nana* Lour.), where paraquat continuously has been used to manage goosegrass for 20 years. The S biotype was collected from the campus of South China Agricultural University (113°36′E, 23°16′N) where no pesticides used. The R biotype was confirmed prior to performing experiments, and the level of resistance in the R biotype was 59.48-fold higher than that in the S biotype [23]. For both biotypes, goosegrass seeds were removed seed-coat with sandpaper, sterilized for 10 min in 3% NaClO, washed 3–5 times and imbibed for 24 h in double-distilled water, germinated in plastic boxes (22×15.5×7 cm) containing a 2:2:1 mixture of soil:peat:sand in a growth chamber at 34°C/28°C (day/night) with a 12 h photoperiod and a light intensity of 800±200 μE m^−2^ s^−1^.

Seedlings of both the S and R biotypes of goosegrass were transplanted into separate plastic 4×24 pots (9×7 cm) with each pot containing 4 plants at 14th day after sowing (S1 Fig), and were sprayed with 0.6 kg·ai ha^−1^ paraquat (Syngenta Corporation, Shanghai, China) (the recommended rate in manufacturer's instructions) using a 3WP-2000 spray tower (Nanjing Research Institute for Agricultural Mechanization, Ministry of Agriculture, Nanjing, China) at 21st day after sowing. Treatment at 0 min represents the absence of paraquat spray as a control, which is no paraquat at this time. Treatments at 30, 60, 90, 120, and 180 min represent the S and R biotype seedlings after spraying paraquat for 30, 60, 90, 120, and 180 min. At each time after treatment, the aboveground parts of S and R biotypes goosegrass in every pot were collected separately to get 8 samples each. And then 6 random samples were selected from every 8 samples with three of them separately for HPLC experiment, but the other 3 samples were mixed as one sample for Real-time PCR experiment. These samples were collected with sterilized scissors and transferred into 10 ml centrifuge tubes using sterilized tweezers. All samples were flash frozen in liquid nitrogen and stored at −80°C pending analysis. Thus, each treatment had 3 biological replicates in both biotypes for HPLC experiment and had 1 mixed biological replicate with 3 technical replicates for Real-time PCR experiment, respectively.

### RNA extraction, cDNA prep and differential expression using RT-PCR

Total RNA of 12 mixed samples (S0, S30, S60, S90, S120, S180 and R0, R30, R60, R90, R120, R180.) was isolated using a EasyPure Plant RNA Kit (TransGen Biotech,Beijing,China). The concentration and purity of total RNAs were evaluated by ultraviolet (UV) light absorption spectra and the A260/A280 ratio (Appendix 1). Total RNA was used to synthesize cDNA with a PrimeScript II 1st Strand cDNA Synthesis Kit (D6210A; TaKaRa, Dalian, China) according to the manufacturer’s protocol. RT-PCRs were carried out using a Bio-Rad iCycler (CFX96, Bio-Rad, Santa Rosa, California, USA) with the following cycling conditions: 95°C for 30 s (20°C s^-1^) and 45 cycles of 95°C for 5 s (20°C s^-1^) and 60°C for 20 s (20°C s^-1^), followed by one cycle of 95°C for 0 s (20°C s^-1^), 60°C for 15 s (20°C s^-1^), and 95°C × 0 s (0.1°C s^-1^). Data were analysed with Bio-Rad CFX Manager Software 1.6 using the 2^−ΔΔCt^ (threshold cycle values) method. Relative PCR product levels were calculated from amplification and dissolution curves for each putative gene. The EST database was searched using the Basic Local Alignment Search Tool (BLAST) [24]. Multiple sequence alignments of amino acid sequences were conducted using CLUSTALW [25]. The high-throughput sequencing results for the 4 selected putative genes (*PqE*, *PqTS1*, *PqTS2*, and *PqTS3*) associated with paraquat resistance in goosegrass were obtained from a previous study [13]. The goosegrass *Actin* gene was amplified as an internal control. The gene-specific primers used for RT-PCR were designed by the software Primer 5 (Primer Premier v5.0, Primer company, Canada) for regions of CDS (Coding sequence) sequence in transcriptome sequence of both biotypes goosegrass with higher variability in Table 1.

**Table 1:** Primers used to amplify four putative genes by real-time PCR.

Gene	Primer	Amplicon length
*PqE*	F:5-GAACAGGCAGTTGGACAC R:5-GTCAGCACGGAGAACATC	85 bp
*PqTS1*	F:5-TTGGTGCTGTTGCTACTT R:5-AATCATCCTCCTCATTATCATACT	81 bp
*PqTS2*	F:5-GTCACAACATACAACAAGAA R:5-GCGAGATACACAACTAAGA	107 bp
*PqTS3*	F:5-CACAATGAACTGATACAAG R:5-TGAACTGATGAAGAGAAC	100 bp
*Actin*	F:5-AACATCGTTCTCAGTGGTGG R:5-CCAGACACTGTACTTCCTTTCA	101 bp

*Actin*: Gene used for internal control.

### HPLC analyses of endogenous polyamines

Endogenous polyamines in the S and R goosegrass biotypes sprayed with paraquat were quantified as described by Flores et al [26]. To extract polyamines, 0.5 g goosegrass leaf tissue was homogenized in 3 ml 5% perchloric acid (PCA) and extracted on ice for 1 h. After centrifugation at 12000 *g* for 20 min at 4°C, the supernatant was collected for analysis using a Varian HPLC system (HPLC; LC-20A, Prominence Series, Shimadzu, Kyoto, Japan). The system comprised a Prostar 210 solvent delivery module, a Prostar 325 UV-Vis Detector, and a 20 μL sample loop (Rheodyne, Rohnert Park, CA, USA). An analytical standard of paraquat for chromatographic analysis was purchased from Sigma Aldrich (St Louis, MO, USA). For each sample, a 100 μL aliquot was analysed for polyamine content after the addition of an equal volume of 5% (w/v) PCA containing 0.12 mM 1,6-diaminohexane (DAH) as an internal standard. A reference solution containing putrescine, spermidine, and spermine was prepared as described above and analysed to establish retention times and signal intensities for each compound and the internal standard. The measurements of polyamine contents in goosegrass were performed according to Wang et al [27].

### Data analysis

Data were analyzed by analysis of variance (ANOVA) at a 95% confidence level using Excel 2010 and SPSS 17.0 (SPSS Inc., Chicago, IL, USA). When the ANOVA indicated significant differences between treatments, means were separated using Duncan’s test at P = 0.05. Data from RT-PCR were divided into two groups as data of S0, S30, S60, S90, S120, S180 and R0, R30, R60, R90, R120, R180. Analyzing effect of comparative data on response different times is of the same biotype in the same treatment in each group. Relative expression of samples was calculated from the value 2^^-ΔΔCt^ based on data of S0 sample as the control. Data from HPLC were analyzed by Processing System (DPS 7.05, Zhejiang, China) which was using completely random design and statistical analysis of single factor test. The model was still Duncan’s test at P = 0.05. The value of Peak area of each sample was calculated that the ratio of peak area of each sample was divided by peak area of the internal standard (1,6-hexanediamine). Relative level of polyamine was equal to the ratio of peak area of each sample divided by peak area of the control sample (S0). Total amount of polyamine (ug/g) was the sum of putrescine content, spermidine content and spermine content.

## Results

### Characterization of four putative genes encoding polyamine transporters

The 4 genes we selected and named *PqE*, *PqTS1*, *PqTS2*, and *PqTS3* in this study have the highest value of the fold change and reads per kilobase of exon model per million mapped reads (PRKM), which indicated that *PqE*, *PqTS1*, *PqTS2*, and *PqTS3* were more closely related to the paraquat resistance in goosegrass from transcriptome analysis work. Therefore, in this study the characterization of *PqE*, *PqTS1*, *PqTS2*, and *PqTS3* was also performed by aligning homogenous sequences from the NCBI website. *PqE*, *PqTS1*, *PqTS2*, and *PqTS3* are reported based on BLASTN search to share high homology (86%) with the *KCS*, *SYP121*, *ABCB*, and *Ca*^*2+*^*-ATP* found in foxtail millet (*Setaria italica* (L.) P. Beauv.), respectively, as shown in Table 2. The foxtail millet is consulted as the closest species with goosegrass in bioinformatics according the BLASTN search on NCBI website without whole-genome sequencing in goosegrass.

**Table 2:** Biological analysis of four putative genes*.

Gene	Homologous gene	Accession	Description	Query cover	E value	Identity
*PqE*	*KCS*	XM_012848229.1	3-ketoacyl-CoA synthase 11-like	99%	0.0	88%
*PqTS1*	*SYP121*	XM_004981451.4	syntaxin-121	87%	0.0	88%
*PqTS2*	*ABCB*	XM_004969589.2	ABC transporter B family member 4-like	90%	0.0	86%
*PqTS3*	*Ca*^*2+*^*-ATP*	XM_004985233.3	calcium-transporting ATPase 1 or plasma membrane-type	81%	0.0	87%

*More information on the KCS, SYP121, ABCB, and Ca2+-ATP genes can be found on the NCBI website (http://blast.ncbi.nlm.nih.gov); it should be noted that their sequences were used as query sequences for BLASTN searches.

### Transcript levels of putative polyamine transporter genes after paraquat treatment

**Fig 1:**
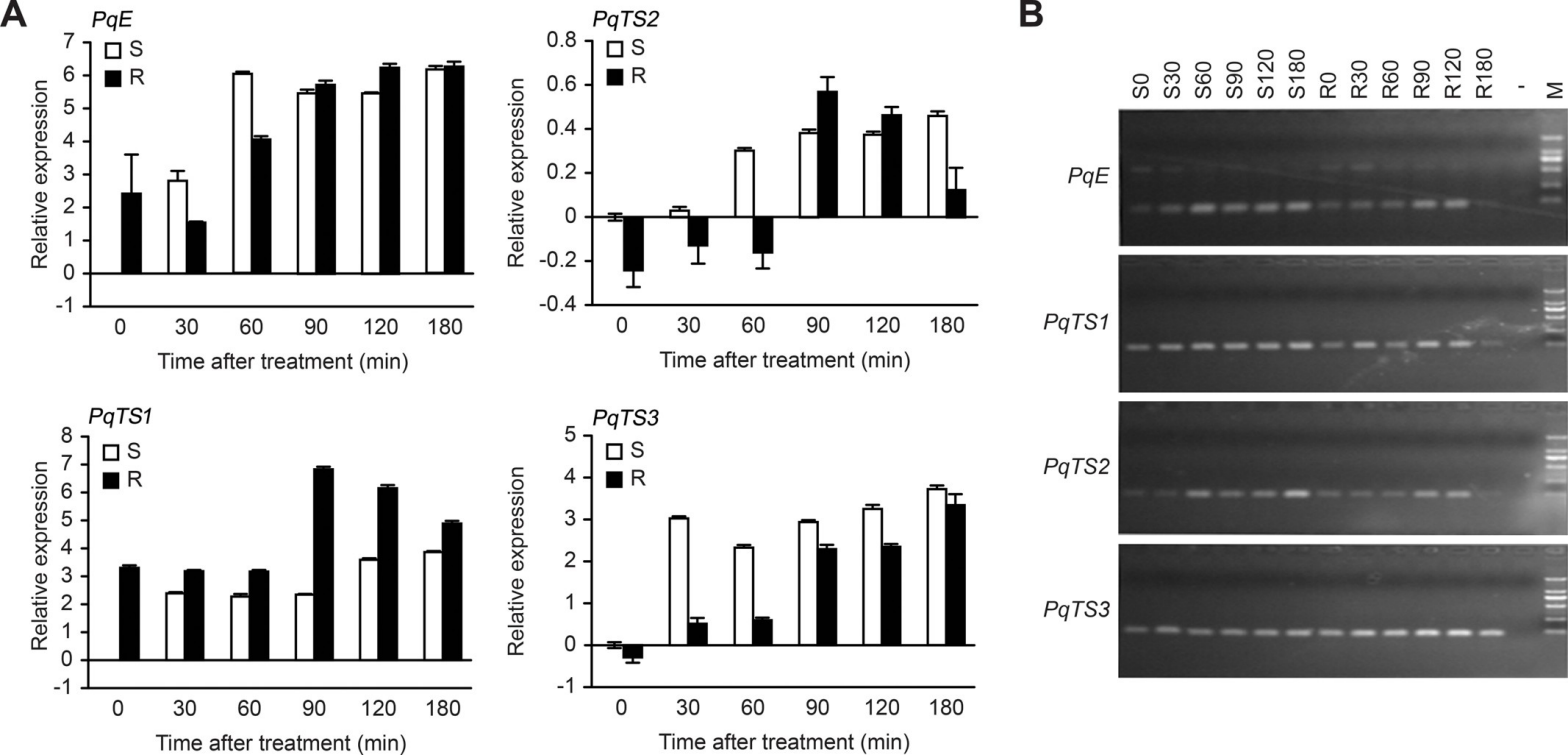
Relative transcript levels of putative genes after paraquat treatment. **A**. Gene transcript levels in susceptible (S) and resistant (R) goosegrass at the indicated times after paraquat treatment. Transcripts were detected by real-time PCR using total RNA extracted from 10-d-old seedlings sprayed with 10/HM paraquat. Error bars represent the SD (n = 3). **B**. Amplification products of 4 putative genes from goosegrass by RT-PCR. For the M:DL 2000 marker, “-” indicates the negative control. R0, R30, R60, R90, R120, and R180 indicate samples of R goosegrass seedlings taken 0, 30, 60, 90, 120 and 180 min after paraquat treatment, while S0, S30, S60, S90, S120 and S180 indicate samples of S goosegrass seedlings taken 0, 30, 60, 90, 120 and 180 min after paraquat treatment, respectively.

The S and R biotypes of goosegrass were treated with paraquat, and the steady-state transcript levels of the selected genes were then determined by RT-PCR. All four putative genes showed different transcription patterns between the S and R biotypes following paraquat treatment (Fig 1A), and the amplification products were of the expected size (Fig 1B).

The S biotype of goosegrass showed an increase in *PqE* transcript levels 0 min to 90 min after paraquat treatment, but the R biotype of goosegrass did not show an increase in *PqE* transcript levels until 60 min after paraquat treatment. At 0 min, the transcript level of *PqE* in the R biotype was 2.4 times higher than that in the S biotype. Thirty minutes after treatment, *PqE* was significantly upregulated in the S biotype but downregulated to the minimum relative expression in the R biotype. Sixty minutes after treatment, the *PqE* transcript level was remarkably increased in the S biotype (6-fold higher than that in the control), whereas it increased to a lesser extent in the R biotype (4-fold higher than that in the control). Ninety minutes after treatment, the transcript level of *PqE* did not differ significantly between the S and R biotypes (5.54-fold and 5.73-fold higher than that in the control, respectively). Finally, stable expression of *PqE* was detected in both ecotypes 120 min and 180 min after paraquat treatment.

The transcript level of *PqTS1* was higher in the R biotype than in the S biotype at each stage 0–180 min after paraquat treatment. This level remained steady in the R biotype until 60 min after paraquat treatment, peaked at 90 min, and then decreased at 120 and 180 min. In the S biotype, the transcript level of *PqTS1* remarkably increased at 30 min; thereafter, it remained stable until 90 min and then increased slightly until 180 min after the paraquat treatment.

The *PqTS2* transcript level showed detectable changes in both the R and S biotypes. The *PqTS2* transcript level was approximately 0.24-fold lower in the R biotype than in the S biotype at 0 min without paraquat treatment. The *PqTS2* transcript levels were upregulated in the S biotype and downregulated in the R biotype 60 min after paraquat treatment. However, 90 and 120 min after paraquat treatment, the *PqTS2* transcript level was higher in the R biotype than in the S biotype.

The paraquat treatment increased *PqTS3* in both biotypes. At 0–90 min after paraquat treatment, *PqTS3* was higher in the S biotype than in the R biotype. To be more specific, although the transcript level of *PqTS3* in the R biotype was 0.32-fold lower than that in the S biotype at 0 min, it steadily increased in the R biotype after 30 to 180 min. In the S biotype, *PqTS3* transcript levels remained relatively steady 30 to 180 min after paraquat treatment.

### Endogenous polyamine contents in goosegrass biotypes

**Fig 2:**
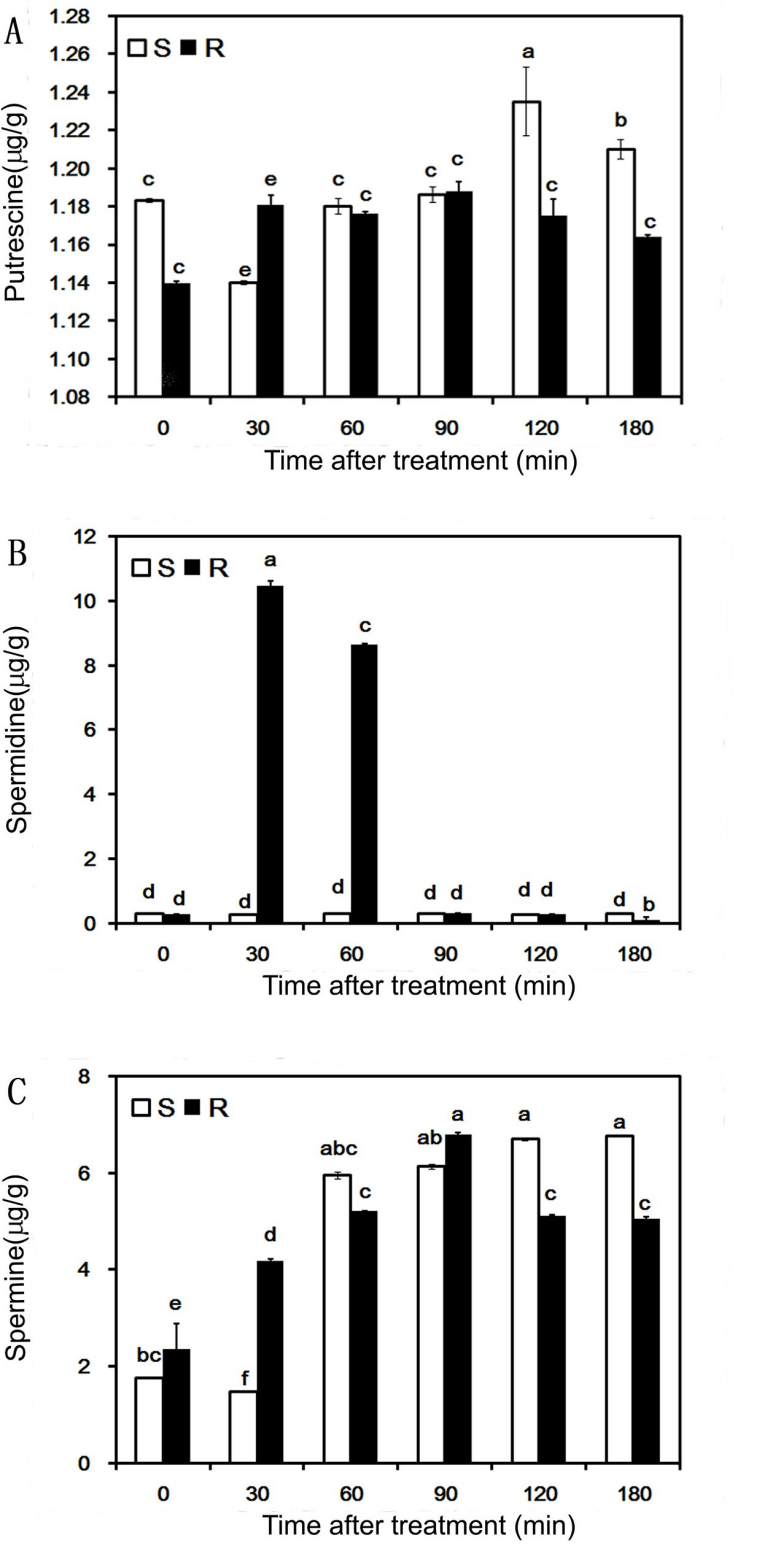
Polyamine levels in susceptible (S) and resistant (R) goosegrass after a paraquat treatment. Putrescine (A), spermidine (B), and spermine (C) were extracted from shoots of R goosegrass (black bar) and S goosegrass (white bar) seedlings 0, 30, 60, 90, 120, and 180 min after spraying with paraquat and then quantified by HPLC. Different lowercase letters indicate significant differences at P<0.05 (t-test).

We used HPLC to detect differences in endogenous polyamine contents between S and R seedlings 0 to 180 min after paraquat treatment (S2 Fig). Polyamine levels in S goosegrass and R goosegrass after a paraquat treatment are shown (Fig 2). In the S biotype, the putrescine content increased after 120 min more than content in 0 to 90 min after paraquat treatment. The spermidine keeps lower content under 2ug/g from 0 to 180 min after paraquat treatment but the spermine contents show significant increase from 60 t0 180 mine after paraquat treatment. In contrast, there were significant changes in polyamine contents, especially spermidine content, in R seedlings after paraquat treatment. After paraquat treatment, the spermidine concentration in R seedlings increased 421.04-fold at 30 min and 346.14-fold at 60 min, but at 90 min, it decreased to an infinitesimal level. In the R biotype, putrescine levels increased 30 min after paraquat treatment and remained steady until 180 min. The spermine content significantly increased in R seedlings 0 to 90 min after paraquat treatment and then decreased 90 to 180 min after treatment. Overall, the Total amount of endogenous polyamines content in the R biotype was higher than that in the S biotype from 0 to 90 min after paraquat treatment in S1 Table, which is covering the time period of the relatively significant difference in contents among the three polyamines.

## Discussion

Paraquat and polyamines show similar uptake characteristics in animal and plant systems, suggesting that paraquat uptake is mediated by polyamine transporters [28]. Fujita et al. also identified an transporter RMV1 to be responsible for uptake of polyamines and its analog paraquat [28]. Paraquat transportation into vacuoles is likely caused by the structural similarities between polyamines and paraquat for their distance in positively changed nitrogen atoms at physiological pH [29,30]. And pre-exposure to putrescine make the resistant biotype in response to paraquat similarly to a susceptible biotype weed *Lolium perenne* L. spp. *Mutiflorud* (Lam) Husnot after evaluating membrane integrity [29].Paraquat toxicity in plants may result when paraquat is taken up by cells via a polyamine transporter with a function under stringent negative regulation by spermidine [31]. Moreover, Kurepa et al. reported that spermidine was the most effective polyamine in protecting *Arabidopsis* against paraquat toxicity, possibly because paraquat is taken up by cells via a polyamine transporter with a function negatively regulated by spermidine [31]. This speculation is based on the fact that paraquat exists as a divalent cation that is structurally similar to *α*,*ω-*diamines and is taken up by cells via polyamine transporters in aqueous solution.

*PqE* shares high homology with the *β-ketoacyl CoA synthase* (*KCS*) gene, which catalyses an essential reaction in the fatty acid elongation process. *PqTS1* shares high homology with *SYP121* based on the BLASTN searches. *SYP121* is reported that it encodes a protein involved in the transport of secretory vesicles at the plasma membrane [32]. Transgenic plants overexpressing *MerC-SYP121* in the plasma membrane were more resistant to cadmium than was the wild type [33]. We found that *PqTS2* shares high homology with *ABCB*, which encodes a member of the ABC transporter B family (ATP-binding cassette) of proteins. This finding suggests that *PqTS2* has functions similar to those of the homogenous *MDR1*/*ABCB1* gene, which is also function at an efflux transporter of paraquat in protection against subsequent toxicity in humans and mice [34]. *PqTS3* shares high homology with Ca^2+^-ATP, which encodes ATPase 1 or plasma membrane-type Ca^2+^-ATP. Paraquat inhibited Ca^2+^ influx across the plasmalemma, and the phenylacetic acid transport system responsible for the movement of paraquat across the plasmalemma was relatively specific to divalent cationic molecules with a distinct charge distribution [35].

Our research corroborates the results of previous studies showing that paraquat treatment can decrease the relative expression level of *PqE* [13]. The *PqE* transcript levels decreased while the spermidine and spermine contents significantly increased in the R biotype after paraquat treatment (Figs 1 and 2). This result may be because paraquat was bound to the polyamine transporter, thereby hindering polyamine transport. The resulting accumulation of polyamines could then reduce the activities of polyamine synthases. Thus, *PqE* might be involved in paraquat resistance and participated in the regulation of endogenous polyamine content. Secondly, *PqTS1* showed higher transcript levels and more sustained expression in the R biotype than in the S biotype after the paraquat treatment. Increases in spermidine and spermine contents alleviated the toxic effects of paraquat in R goosegrass 0 to 60 min after spraying, suggesting that *PqTS1* played a role in vesicular membrane transport of spermidine and spermine in cells. Thus, the upregulation of *PqTS1* expression was an important part of the resistance response to paraquat in goosegrass. However, further studies on *PqTS1* are required to clarify its role in paraquat resistance. Thirdly, the transcript level of *PqTS2* in the R biotype decreased 0 to 60 min after the paraquat treatment, and the subsequent detoxification effect of spermidine decreased 90–120 min after spraying with paraquat, possibly indicating that the increased expression of *PqTS2* 90 min after the paraquat treatment affected paraquat transport from goosegrass leaves. The concentration of putrescine did not differ significantly between the R and S biotypes 0–60 min after the paraquat treatment. This result was consistent with the finding that paraquat completely inhibited putrescine uptake but not spermidine uptake by the spermidine-preferential ABC transporter in *E*. *coli* [36]. The spermidine content was closely related to the *PqTS2* transcript levels in goosegrass in our study, suggesting that *PqTS2* plays a key role in the molecular mechanism of paraquat resistance. However, further research is required to explore the function of spermine in paraquat resistance. Finally, the relative expression of *PqTS3* was significantly different between the S biotype and R biotype after paraquat treatment in our study. Based on this finding, we speculate that the putative gene *PqTS3* takes part in the regulation of the levels of spermidine and spermine to alleviate paraquat toxicity in R goosegrass, which could lead to later signal reception of paraquat in R goosegrass than in S goosegrass.

## Conclusion

Endogenous polyamines actually play a vital role in paraquat resistant in goosegrass, especially the spermidine. Meanwhile, four genes have been identified in this paraquat-resistance weed, which directly or indirectly involved in encoding putative components of polyamine transport and synthesis systems.

## Supporting information

S1 Table Paraquat effects on the endogenous polyamine contents in goosegrass. (DOCX)

S1 Fig The growth of susceptible (S) and resistant (R) goosegrass biotype for designed experiments on various time after treatments of paraquat. Time at 0 min labelled samples in collection for use without paraquat as a control. Time at 30, 60, 90, 120, and 180 min labelled samples in collection for use after spraying paraquat for 30, 60, 90, 120, and 180 min. RT-PCR and HPLC labelled samples for use in corresponding experiments.
(JPG)

S2 Fig HPLC wave peak diagram of polyamine contents in susceptible (S) and resistant (R) goosegrass after paraquat treatment. R0, R30, R60, R90, R120, and R180 indicate samples of the resistant goosegrass seedlings taken 0, 30, 60, 90, 120 and 180 min after paraquat treatment, while S0, S30, S60, S90, S120 and S180 indicate samples of susceptible goosegrass seedlings taken 0, 30, 60, 90, 120 and 180 min after paraquat treatment, respectively. Put represents putrescine, Spd represents spermidine, and Spm represents spermine.
(TIF)

S3 Fig (TIFF)

S1 Data (XLS)
