# Supplementary material for: Differences of endogenous polyamines and putative genes associated with paraquat resistance in goosegrass (Eleusine indica L.)
Source: PLoS One. 2019 Dec 26;14(12):e0216513. doi: 10.1371/journal.pone.0216513 (PMC6932794; doi:10.1371/journal.pone.0216513)
Supplement: S1 Table — (DOCX) [file pone.0216513.s001.docx]

**S1 Table. Paraquat effects on the endogenous polyamine contents in goosegrass.**

| Type | Polyamine content (µg g·FW^-1^) | | | | | |
| --- | --- | --- | --- | --- | --- | --- |
|  | 0 min | 30 min | 60 min | 90 min | 120 min | 180 min |
| Susceptible (S) goosegrass | 32.48i | 28.84j | 74.08e | 76.05d | 82.11c | 82.81c |
| Resistant (R) goosegrass | 37.79h | 158.24a | 150.36b | 83.04c | 65.73f | 63.24g |

Polyamines: Putrescine, spermidine and spermine. Different lowercase letters indicate significant differences at P<0.05 (t-test).
