# Supplementary figures and images for: Differences of endogenous polyamines and putative genes associated with paraquat resistance in goosegrass (Eleusine indica L.)
Source: PLoS One. 2019 Dec 26;14(12):e0216513. doi: 10.1371/journal.pone.0216513 (PMC6932794; doi:10.1371/journal.pone.0216513)

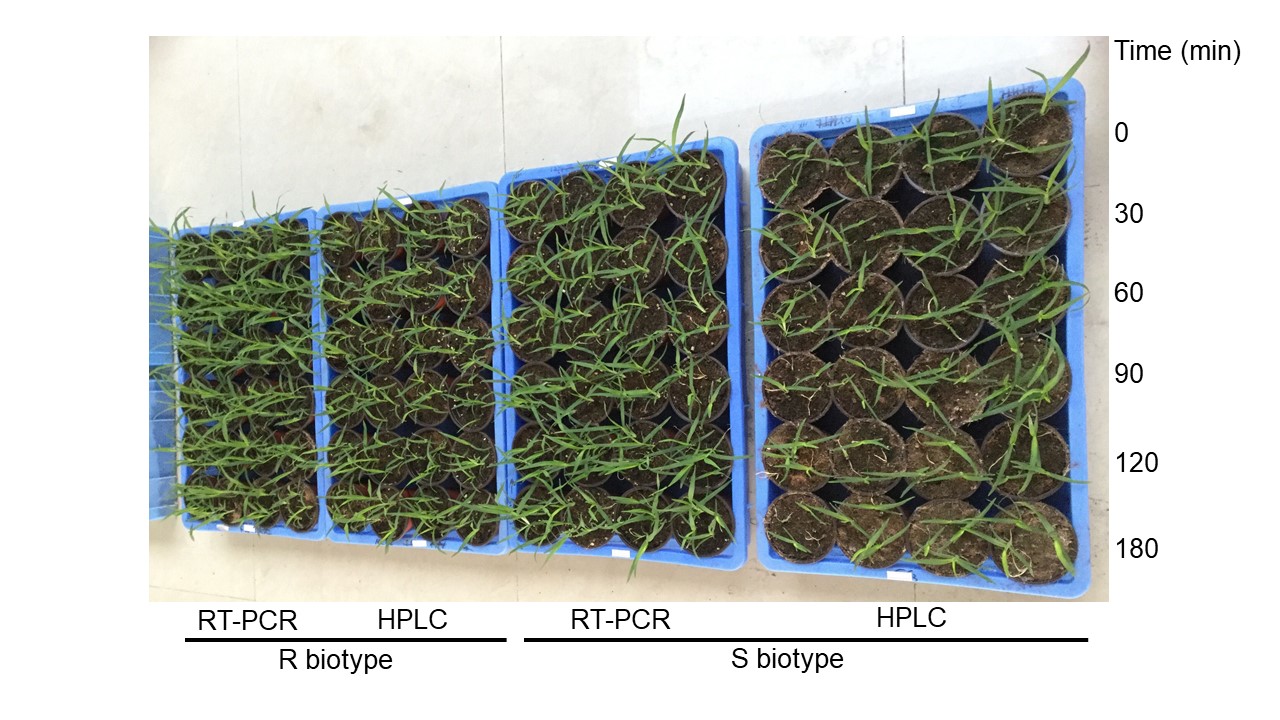

Supplement: S1 Fig — Time at 0 min labelled samples in collection for use without paraquat as a control. Time at 30, 60, 90, 120, and 180 min labelled samples in collection for use after spraying paraquat for 30, 60, 90, 120, and 180 min. RT-PCR and HPLC labelled samples for use in corresponding experiments. (JPG) [file pone.0216513.s002.jpg]
